# Lipid Nanoparticles: Promising Treatment Approach for Parkinson’s Disease

**DOI:** 10.3390/ijms23169361

**Published:** 2022-08-19

**Authors:** Keelan Jagaran, Moganavelli Singh

**Affiliations:** Nano-Gene and Drug Delivery Laboratory, Discipline of Biochemistry, University of KwaZulu-Natal, Private Bag X54001, Durban 4000, South Africa

**Keywords:** Parkinson’s disease, lipid nanoparticles, drug delivery, blood–brain barrier, nanomedicine

## Abstract

Parkinson’s disease (PD), a neurodegenerative disorder, is a life-altering, debilitating disease exhibiting a severe physical, psychological, and financial burden on patients. Globally, approximately 7–10 million people are afflicted with this disease, with the number of cases estimated to increase to 12.9 million by 2040. PD is a progressive movement disorder with nonmotor symptoms, including insomnia, depression, anxiety, and anosmia. While current therapeutics are available to PD patients, this treatment remains palliative, necessitating alternative treatment approaches. A major hurdle in treating PD is the protective nature of the blood–brain barrier (BBB) and its ability to limit access to foreign molecules, including therapeutics. Drugs utilized presently are nonspecific and administered at dosages that result in numerous adverse side effects. Nanomedicine has emerged as a potential strategy for treating many diseases. From the array of nanomaterials available, lipid nanoparticles (LNPs) possess various advantages, including enhanced permeability to the brain via passive diffusion and specific and nonspecific transporters. Their bioavailability, nontoxic nature, ability to be conjugated to drugs, and targeting moieties catapult LNPs as a promising therapeutic nanocarriers for PD. While PD-related studies are limited, their potential as therapeutics is evident in their formulations as vaccines. This review is aimed at examining the roles and properties of LNPs that make them efficient therapeutic nanodelivery vehicles for the treatment of PD, including therapeutic advances made to date.

## 1. Introduction

Parkinson’s disease (PD) presents itself as a life-altering and debilitating disease that primarily affects the neuronal make-up of the brain. It is deemed a neurodegenerative disorder. It is estimated that 7–10 million people are afflicted with this disease worldwide, with a prevalence rate of 41 in 100,000 people. Notably, the prevalence rate increases to 1900 people per 100,000 in individuals over 80 years old [1]. This growing health issue is postulated to see an increased prevalence to 12.9 million cases by 2040 [2].

Clinically, PD is a progressive movement disorder with various nonmotor symptoms, including sleep disturbance, constipation, depression, anxiety, and anosmia [3,4]. This disease manifests in two forms: (i) sporadic (idiopathic), which is caused by a gene–environment interaction, and (ii) familial, which is genetically inherited in either an autosomal recessive or dominant manner [5,6]. Genetic mutations that cause the disease are noted in some genes, such as the *LRRK2, PARK7, PINK1, PRKN*, and *SNCA*. The resulting manifestation of parkinsonian symptoms is due to a pathological effect, which is observed as loss of dopaminergic neurons in the substantia nigra and presence of various protein aggregates (including α-synuclein), called Lewy bodies, in the midbrain [7].

Current therapeutics are palliative, suggesting the need for a novel efficacious strategy to treat PD. Drugs such as levodopa and ropinirole have been met with challenges, especially their need to cross the blood–brain barrier (BBB). Due to the protective ability of the BBB to resist the permeability of foreign molecules into the brain [8], the drugs that are administered constitute low concentrations of dopamine, which result in several side effects in patients [9,10]. The emergence of nanomedicine with its array of nanoparticles (NPs) has unfolded a new route for therapy. Of these, lipid NPs (LNPs) provide significant advantages regarding improved bioavailability, permeability, and solubility. Furthermore, they exhibit high drug-loading capacity, low cytotoxicity, ease of surface modification, and an ability to permit cell-specific targeting [11]. In a study directed at the treatment of glioblastomas, a multifunctional NP comprising the Nutlin-3a drug and superparamagnetic NPs encompassed by LNPs was used. The results obtained highlighted the natural ability of lipids to effectively cross the BBB and to protect the encapsulated cargo while inducing proapoptosis in glioblastoma cells [12]. Although targeting and therapeutics differ between cancer and PD, the ability of these LNPs to permeate the BBB is an important property to be noted for neurodegenerative disease studies.

LNPs present a potentially effective drug delivery strategy for safe and site-specific delivery of therapeutic agents for treatment of PD. Drug nanocarriers can provide advantages such as increased half-life of the therapeutic, reduction in the drug dosage, and reduction in unpleasant side effects [13]. It is due to these reasons that LNPs have made their way to the forefront of nanomedicine. This review looks at the genetics that govern PD, the NPs being used in nanomedicine, and the potential benefits of using LNPs as therapeutic nanodelivery vehicles and their ability to cross the BBB.

## 2. Parkinson’s Disease

James Parkinson first described a highly complex, progressive neurodegenerative disorder, aptly named Parkinson’s disease (PD). While explaining this disorder as a “shaking palsy”, he also highlighted the urgency to mitigate this disorder, stating “there appears to be sufficient reason for hoping that some remedial process may ere long be discovered, by which, at least, the progress of the disease may be stopped” [14]. Despite the advancements in medicine, there has yet to be a cure for PD.

PD falls primarily under neurodegenerative disorders that affect neurons of the human brain, resulting in deterioration of the brain function. This debilitating disease is unfortunately incurable, with palliative therapeutics being administered to treat the symptoms. Based on their characteristics, neurodegenerative disorders can be broadly divided as having selective neuronal or regional vulnerability. The former occurs due to the disease pathology affecting particular neurons, while the latter is the deterioration of the pathology over time, impacting a greater number of regions predictably and stereotypically [15].

### 2.1. Neuropathological Hallmarks of Parkinson’s Disease

Understanding a disease’s neuropathological hallmarks is imperative in developing an appropriate treatment strategy. The onset of the disease occurs in the substantia nigra (SN) pars compacta in the midbrain and begins with degeneration of the dopaminergic neurons and protein aggregates known as Lewy bodies. These protein aggregates are noted as cytoplasmic inclusions together with insoluble aggregates of alpha-synuclein [16]. Autopsies of patients with PD have shown α-synucleinopathies and tauopathies, corticobasal degeneration (CBD), and progressive supranuclear palsy (PSP) as the most common causes of parkinsonism [17].

Selective neuronal vulnerability is eminent in PD and can be inherited or sporadic. Within the SN, two cell groups are affected: the medial and dorsal cell groups (A10 or mesolimbic pathway) are resistant, while the ventrolateral cell groups (A9 or nigrostriatal pathway) are vulnerable. This vulnerable state is linked to calcium transients, where deficient calcium buffering occurs in A9 compared to A10 neurons, allowing the former dopaminergic neurons to remain vulnerable to cellular stress [17]. Furthermore, a significant reduction in the integrity of the nuclear membrane is noted, which leads to the release of proaggregant nuclear factors that trigger α-synuclein aggregation. Following the aggregation, the spread to other cells is either direct or indirect, leading to parkinsonian symptoms [18].

Another key indication of the development of PD is the overproduction and inability to effectively detoxify reactive oxygen species (ROS) and reactive nitrogen species (RNS) [19]. Oxidative and nitrative stress promote the degeneration of dopaminergic neurons in PD. This causes the disruption of important biological processes, resulting in cellular demise [20]. Following the disruption of these key components in the PD substantia nigra, dysregulation of iron and calcium metabolism, increase in neuroinflammatory cells, aging, and mitochondrial dysfunction are imminent [21].

An interesting early patient-based study reflected on the presence of accumulated blood RNA biomarkers in PD. The process of nonsense-mediated decay (NMD) was reported to degrade mRNA and play a regulatory role in the brain. The authors proposed the use of deep brain stimulation surgery to modulate NMD of RNA in the leukocytes of Parkinson’s patients and improve the motor-related symptoms associated with PD [22].

### 2.2. Clinical Manifestations and Determinants of Parkinson’s Disease

While the mechanism for the onset of the disease is understood, clinical motor symptoms are only presented following the death of 50–70% of SN dopaminergic neurons, suggesting the need to devise a means of identifying the cause before physical manifestations develop [16]. The motor symptoms include muscle tone rigidity, postural instability, bradykinesia, and resting tremors. Beyond this, nonmotor symptoms may also be seen in patients succumbing to PD, such as dementia, autonomic dysfunctions, sleep disorders, sensory abnormalities, depression, and anxiety [23].

Similar to cancer, the onset of PD may be due to environmental or genetic factors. Factors such as head injuries or exposure to toxic chemicals may significantly increase a person’s susceptibility to PD. While environmental factors play a crucial role in PD, they can also further trigger patients who are already genetically predisposed to the disease. This was noted in a study on monozygotic and dizygotic twins. The comparison of the concordance rates, which estimated the heritability rate of PD, was found to be 30%, indicating that most PD risk is related to behavioral and environmental factors [24].

Environmental or external factors that pose a risk to individuals predisposed to PD include, but are not limited to, vigorous exercise, plasma urate, smoking, ibuprofen, and high consumption of coffee. Beyond this, certain pesticides and trauma to the brain have also been recognized as determinants of PD [25]. Further studies have provided greater insight into pesticide exposure and its positive correlation to PD onset in farm workers and rural residences. Laboratory studies have portrayed the use of several dithiocarbamates, rotenone, organochlorines, paraquat, and 2,4-D as causative agents in PD [26,27]. It has further been observed that mild to moderate head injuries, which may have occurred decades before disease onset, are associated with greater risk of PD. The number of injuries and the positioning of the trauma, together with genetic susceptibility, was proposed to increase the risk two- to five-fold [27].

Genetic mutations in the encoded protein that lead to PD disease are either familial or sporadic (gene–environment interactions). Table 1 summarizes the various genes involved in PD together with their respective mechanisms of action. Despite this information, much remains unknown, warranting further in-depth studies. Treating the cause of PD at a genomic level may retard the degeneration of many dopaminergic neurons.

### 2.3. Current Therapeutics

**Figure 1:**
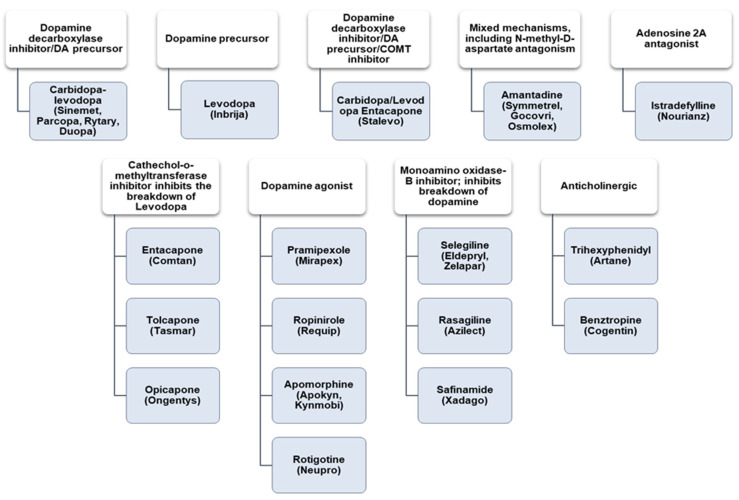
Commonly used drugs (shaded) are grouped with their functions (white) and PD therapeutics. Adapted from [33].

Because the current treatment of PD remains palliative, a cure lies in treating the primary causes, such as genetic defects or mutations. To date, dopaminergic administration has been effective for short periods in movement disorders, while antipsychotic medications treat the psychosomatic symptoms [33]. Figure 1 summarizes the currently utilized medications for PD treatments and their functions. The major drawback to these medications is their poor ability to efficiently permeate the blood–brain barrier (BBB), causing their localization in the CNS. This often results in low-dose concentrations being administered [34].

As a result of the previously mentioned challenges to the current drugs employed, it is imperative to seek alternative avenues to close the gap between palliative treatment and a cure. One such approach can be the integration of nanomedicine into therapeutic delivery to the brain.

## 3. Nanomedicine

Nanomedicine is known as the utilization of nanosized particles in health and medicine. It is a revolutionary novel system that explores an alternate avenue in treating diseases with greater specificity and efficiency. Some NPs can provide a theranostic approach to medicine, with this duality being a significant advantage [35]. The inexpensive means of creating these NPs, together with the ability of their surfaces to be easily manipulated for different tissue targets, has enhanced their importance in medicine. Several studies have demonstrated the potential of nanomedicine in treating diseases where traditional medicine had failed. The amalgamation of current advances in biology, material science, chemistry, and physics to aid diagnostic and treatment strategies is now coming to fruition [36]. To date, many different NPs have been synthesized and used in nanomedicine, with novel NPs regularly evolving to add to the arsenal of NPs at our disposal. This improves the chances of treating a wide range of disorders as each NP possesses its unique properties and can be tailor-made to treat a specific disease. Generally, NPs can be classed as being inorganic, organic, or carbon-based NPs (Table 2).

The principal use of NPs is to overcome challenges faced by commonly used drugs, such as poor stability, potential immunogenicity, solubility, and reduced plasma half-life [37]. Nanodelivery systems can increase the therapeutics circulation time, allow several different administration routes, circumvent potential solubility issues using hydrophobic molecules, and cater for favorable biodistribution of the therapeutic gene or drug [38]. Overall, their ideal size, quantum properties, ability to be conjugated to pharmacologically active agents, and favorable surface-to-mass ratio assures their potential as therapeutic delivery systems [39]. Nanoscale particles (<100 nm) favor the passage through biological barriers, such as those found in the nervous system, lung, and vasculature surrounding tumors [38,40]. Nanomaterials have shown the ability to facilitate the stability and protection of genetic materials such as DNA, mRNA, and siRNA and to enhance transfection efficacy with low cytotoxicity [41,42,43]. Clinical trials have since been conducted for cancer and fungal infections, utilizing liposomes to deliver doxorubicin and amphotericin B [37]. One of the optimistic outcomes of the application of nanomedicine involves using brain tumor targeting for efficient passage across the BBB [8,44]. The current review will discuss the organic class of NPs and focus on lipid NPs (LNPs).

### 3.1. Lipid Nanoparticles

**Figure 2:**
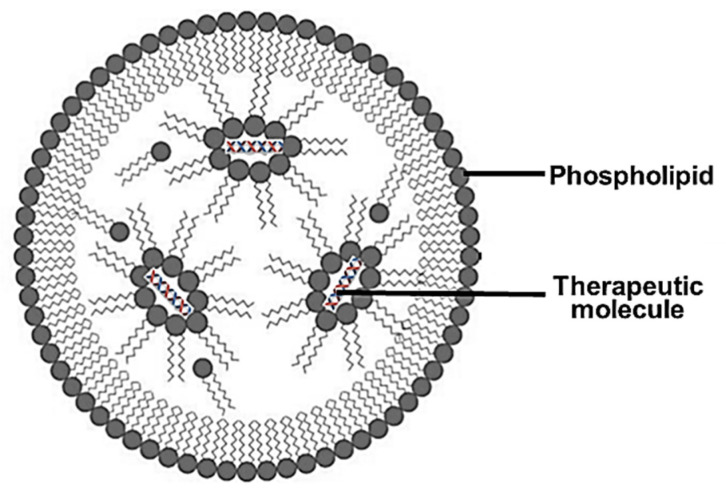
An illustration of a lipid nanoparticle showing the outer phospholipid layer and the encapsulated therapeutics.

Lipid NPs (LNPs) have been the most popular NPs with regard to progress into clinical trials, possibly due to their lipophilic, bioacceptable, and biodegradable nature, which permits a less toxic therapeutic approach. These LNPs have shown promising results as therapeutic delivery vehicles and have gained popularity since their use as a delivery vehicle of mRNA in the COVID-19 vaccine. The lipids were able to house the mRNA in vivo while remaining stable in the bloodstream before being taken up by phagocytic cells via endocytosis. This LNP acted as an immunological adjuvant to elicit immune responses against the spike proteins of the virus [48]. LNPs (Figure 2) are usually spherical vesicles composed of ionizable cationic lipids and a helper lipid.

LNPs can change charge based on their environment, portraying a neutral charge at physiological pH with low toxicity and a positive charge at low pH that permits nucleic acid complexation. These LNPs possess improved cellular uptake, circulation half-life, and endosomal escape [49]. This is primarily due to the ionizable properties of the lipids at low pH, which permit the release of pharmacologically active agents directly into the cytoplasm. LNPs are easily modified by selecting an appropriate composition of NP, which includes a helper lipid to promote cell binding and uptake. Polyethylene glycol (PEG) has been commonly added to significantly reduce opsonization by serum proteins and to reduce reticuloendothelial clearance. Adding cholesterol also assists by filling the crevices between the lipid molecules, adding stability, and favoring cell membrane fusion [50]. The size of the lipids, surface charge, and the specific lipid used in the formulation will influence the performance of LNPs in vivo.

Zhao and coworkers constructed LNPs that were surface modified to load a basic fibroblast growth factor (bFGF) for targeting the brain via administration through the nasal cavity. The study utilized a gelatin polymer mixed with bFGF as the aqueous phase, followed by the incorporation of hydrogenated soy phosphatidylcholine as the lipid phase. The formulated LNPs portrayed high stability (zeta potential~−27.6 mV), were ~172 nm in size, and had an entrapment efficiency of around 86.7%. This novel therapeutic system was found to cause no adverse effects, had low cytotoxicity, and efficiently transported the active pharmacological agent to the olfactory bulb and striatum in a hemiparkinsonian rat model. The benefit of LNPs was evident when compared to the poor efficiency and stability of naked bGFG [51].

Various lipid nanoformulations have been produced to date. These include liposomes, solid lipid nanoparticles, lipid nanoemulsions, nanostructured lipid carriers, and lipid–polymer hybrid carriers. These will be briefly discussed.

### 3.2. Liposomes

**Figure 3:**
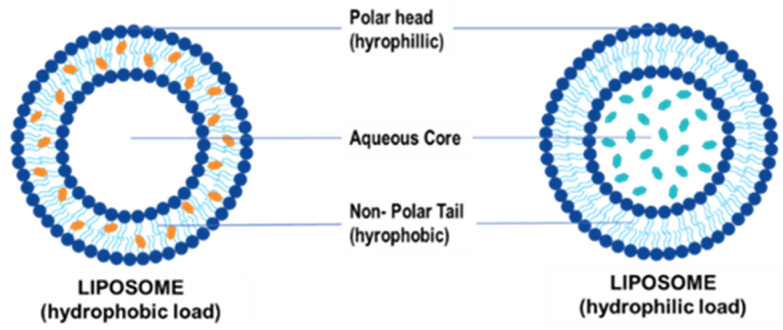
Illustration of a liposome and encapsulation of hydrophilic and hydrophobic molecules.

Liposomes were initially identified in the 1960s following the spontaneous formation of closed lipid bilayers in water [52]. It remains a popular choice for lipid-based vehicles because of its simple structure [53]. These NPs are composed of an aqueous core that encapsulates the drug or gene of choice and is enclosed in a unilamellar or multilamellar phospholipid bilayer that contains both hydrophilic and hydrophobic groups (Figure 3) [54].

Unilamellar liposomes are 20–250 nm, have a single lipid layer surrounding an aqueous core, and are ideal for encapsulating drugs or genes. Multilamellar liposomes are larger in size (1–5 µm) and can have two or more concentric lipid bilayers for entrapping biomolecules [55]. Liposomes have been classed as anionic, neutral, or cationic depending on their lipid composition. Neutral and anionic liposomes generally encapsulate their therapeutic cargo, while cationic liposomes can also electrostatically bind to nucleic acids to produce lipoplexes [53].

Studies over the years have shown that liposomes can effectively protect their genetic cargo in vitro [50,56,57]. This protection ability of liposomes is due to the phospholipid bilayer acting as a barrier to fluctuating pH conditions, enzyme action, and free radicals, thereby preventing degradation of pharmacologically active agents and genetic material before their release [55]. To produce liposomes that are devoid of vesicle surface-to-surface interactions, an ionizable or cationic lipid that improves adjuvanticity, cholesterol for stability of the lipid membrane in vivo, and distearoyl phosphatidylcholine together with pegylated lipids have been used [50,52,56].

### 3.3. Solid Lipid Nanoparticles and Nanostructured Lipid Carriers

**Figure 4:**
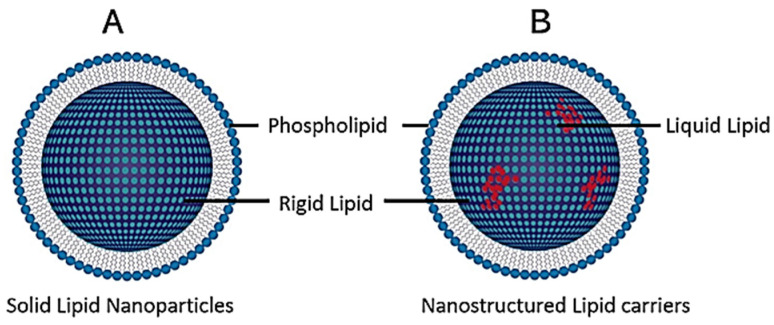
General structure of (**A**) solid lipid nanoparticles (SLNPs) and (**B**) nanostructured lipid carriers (NLCs).

Although liposomes have shown great efficiency and have been the most popular to date, they possess some limitations with respect to low drug entrapment, difficulty in ach performance at a large scale, and the requirement for complex production methods using organic solvents [52]. To this end, solid lipid nanoparticles (SLNs) and nanostructured lipid carriers (NLCs) were developed (Figure 4). The significant difference between these is seen in the crystalline lipid layers, with liposomes comprising liquid crystalline bilayers of the lipid, while SLNs contain lipids that are solid at physiological temperature and NLC are made up of a mixture of both [58,59,60].

Depending on their synthesis, SLNs possess more of an advantage due to their size (40 to 1000 nm) while exhibiting enhanced physical stability. Furthermore, SLNs and NLCs have shown greater bioavailability, higher loading capacities, controlled cargo release, production on a larger scale, and the ability to carry out synthesis without organic solvents [52]. It has been reported that SLNs are taken up by clathrin-mediated endocytosis and degraded in the lysosome to release the therapeutics [60]. The drawback of SLNs is seen in their long-term storage, with the occurrence of crystallization and possible expulsion of the cargo into the storage media [61]. To overcome this occurrence, NLCs have been formulated with solid and liquid lipids at room temperature, significantly reducing the degree of crystallinity [59,60].

## 4. LNPs and the Blood–Brain Barrier

A significant obstacle in developing an efficient therapeutic system for PD is the inability of drugs, peptides, and large molecules to pass through the brain’s endothelial cellular lining, known as the blood–brain barrier (BBB) [62]. When designing appropriate LNPs for traversing the BBB, the brain itself needs to be prioritized to develop a strategy to bypass the physiological mechanisms in place to prevent the entry of foreign substances. Within the structure of the BBB are brain capillary endothelial cells, pericytes, perivascular mast cells, basement membranes, astrocytes, and neuronal cells, which govern the exchange of molecules between the blood and the brain [8,33,63,64]. The brain capillary endothelial cells (BCECs) provide a significant protective ability via their close attachment to each other, creating a tight junction. This tight junction eliminates the risk of harmful toxins and pathogens entering the brain, while the degrading enzymes act as a secondary defense mechanism [8,65].

The BBB’s natural ability to permit the entry of specific molecules can be exploited using LNPs. Research has shown that entry provisions occur via various mechanisms, such as receptor mediation, endocytosis, carrier-mediated transcytosis, cell-mediated endocytosis, adsorptive transcytosis, the transcellular pathway used for small lipoidal compounds, and paracellular diffusion employed for hydrophilic substances [66,67]. The ascendancy of exploiting these natural properties of the BBB creates loopholes for developing novel LNPs due to their unique capabilities that enable them to target and traverse the BBB [68]. LNP surfaces can be enhanced via surface modifications, which allow for effective site-specific targeting [69]. Numerous receptors have been identified on the surface of the BBB, which can effectively be used as surface-active ligands to facilitate receptor-mediated transcytosis [70]. Some of these targeting strategies are outlined in Table 3.

From the ligands in Table 3, the conjugation of Angiopep-2 to both organic and inorganic NPs carrying therapeutic genes or drugs has been studied to treat brain cancer, brain injury, stroke, epilepsy, fungal infections, Alzheimer’s disease (AD), and Parkinson’s disease (PD) [8]. Angiopep-2 has shown the ability for transcytosis and accumulation in the parenchyma. Recently, a phase II study ANG1005 (made up of paclitaxel residues linked to Angiopep-2) produced positive results in patients with breast-cancer-related brain metastases [80]. Appending Angiopep-2 to LNPs may be favorable as it can promote transport across the BBB and shows specificity for glioma cells that overexpress low-density lipoprotein receptor-related protein-1 (LRP1) on their surfaces [81].

Compared to inorganic NPs, LNPs portray several advantages. NPs produced by chemical and physical means incur high manufacturing costs, and the process is time-consuming. Metallic NPs utilizing zinc oxide and copper oxide have been reported to cause toxicity to the environment and the host tissue [82]. In order to circumvent this toxicity, many researchers have turned to biological synthesis methods [83]. LNPs generally have a phospholipid outer layer that physiologically resembles the cellular membrane. This enhances cellular uptake and the possibility of them passing through the BBB [84]. The increased BBB permeability occurs via the P-glycoprotein efflux system, which offers a means to cross the BBB. Furthermore, the efficient encapsulation properties of these LNPs protect the loaded drug or gene from early systemic enzyme degradation [85]. Further protection is offered by the cholesterol component of the lipid carrier. This adds to the retention of natural homeostasis in a biological environment and reduction in the entry of water into the LNPs to avoid premature degradation [84]. This was validated in a study where LNPs were shown to specifically target tumor sites, including the glioblastoma regions [86,87]. A comparable xenobiotic metabolism was noted in LNPs and food-based lipids that were internally degraded into nontoxic residues. [88]. This further highlights their nontoxic nature.

### 4.1. LNPs for Parkinson’s Disease

Lipid-based NPs have been highlighted for vaccination and therapy. Its natural ability to penetrate the brain increases its attractiveness for treatment directed at neurological diseases. These LNPs have emerged as appropriate nanocarriers due to their favorable size, surface charge, tunable surface area, and morphology [89].

**Figure 5:**
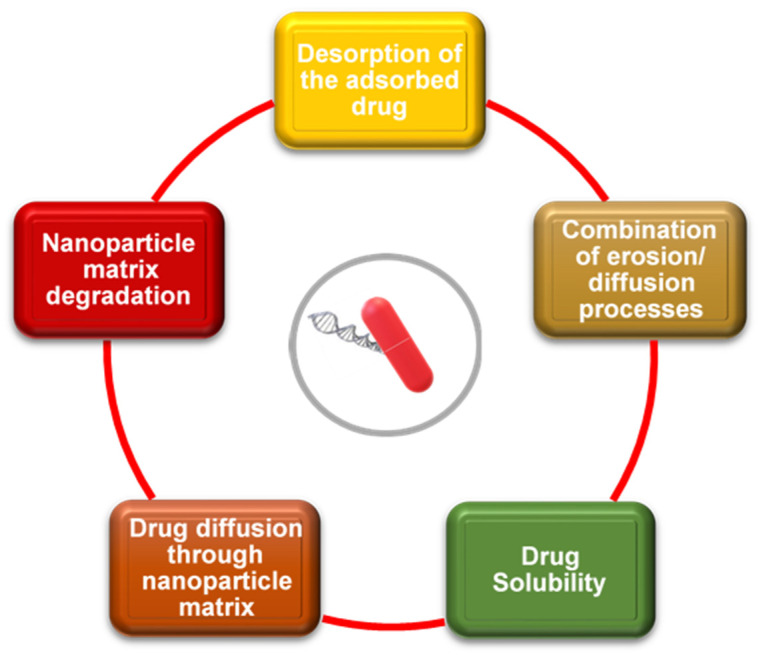
Important factors to consider when designing lipid nanoparticles for drug delivery. Adapted from [90].

The use of nanomedicine in drug delivery has an array of advantages, including increased resistance time in the host (that is, increasing the half-life for clearance), improved bioavailability due to enhanced aqueous solubility, and greater specificity with regard to targeting diseases, such as PD [90]. This reduces the side effects manifesting in nontarget tissues and cells of patients due to the concomitant drop in drug concentration and safe and efficient delivery to the target tissue [13]. When designing a LNP for drug delivery, many factors need to be taken into consideration in order to improve therapeutic indices. Some of these factors are summarized in Figure 5.

When looking at currently available treatments, dopamine supplementation is necessary to compensate for the loss of dopaminergic neurons in PD patients. A recent study utilized an albumin/PLGA NP conjugated to dopamine. The nanocomplexes exhibited successful permeability to the brain by effectively crossing the BBB. This was attributed to the albumin-coated NP, which enhanced the interactions of the NP with specific cell membrane receptors. Furthermore, using dopamine instead of L-DOPA (a drug commonly converted to dopamine in vivo) reduced symptoms in a mouse model compared to control NPs without dopamine and mice administered with L-DOPA. Improvements manifested as restoration of balance, motor coordination, and sensorimotor performance [91].

The efficacy of nanomedicine and drug therapy is noted further in a study conducted by Dudhipala and Gorre (2020), who utilized LNPs conjugated with ropinirole (RP), a dopamine agonist. Increased pharmacokinetics was exhibited with respect to the drug in the host, with more than two-fold enhancement in oral administration, three-fold enhancement in topical administration, and single-fold enhancement in topical bioavailability in SLN and NLC complexes. Pharmacodynamic studies have portrayed increased levels of glutathione, catalase, and dopamine with a reduction in lipid peroxidation levels [92].

Functionalized liposomes containing the dopamine derivative N-3,4-bis(pivaloyloxy)-dopamine, together with a brain-targeted delivery system made up of a 29 amino acid peptides (RVG29) derived from the rabies virus glycoprotein, were studied. Significantly improved cellular uptake was noted in both the endothelial and dopaminergic cells, with improved penetration of the BBB. Furthermore, enhanced therapeutic efficacy was noted due to the RVG29-LNPs being selectively driven to the substantia nigra and striatum [93].

In these two independent studies, the common trend of increased performance with lower side effects was clearly noted, with significant improvements in the parkinsonian symptoms exhibited. While drug therapy is an effective therapeutic, gene therapy is another promising PD treatment strategy. Gene therapy aims to knock down or replace the causative gene/s, as mentioned earlier, to treat PD at the root. Hence, this remains to be fully explored.

### 4.2. An Update on Clinical Trials Using Lipid Nanoparticles

There are some approved treatment strategies available to treat PD symptoms, e.g., transdermal patches. The transdermal patches using rotigotine (Neupro^®^) treats the restless leg syndrome symptoms of PD, while selegiline (Emsam^®^) is used to overcome depression. However, these also come with some noted side effects. [94]. Notably, LNPs are not employed in these formulations. The intravenous therapeutic patisiran (ONPATTRO^®^), which has been approved for treatment of polyneuropathy, utilizes LNPs for the delivery of a therapeutic siRNA [95]. However, a latest search of the NIH library for clinical trials in the last 10 years revealed that LNPs are yet to be exploited as nanocarriers for therapeutics to treat PD. One Phase 1 study using liposomes commenced in 2021 and is scheduled to be completed in December 2022. The study simply evaluates the safety of Talineuren, which comprises GM1 (a monosialotetrahexosylganglioside) as the therapeutic, in combination with a proprietary liposomal formulation [96]. However, one study using gold nanocrystals has been completed [97]. The results are awaited and could signal a new direction for nano-based therapeutics and a milestone for nanomedicine. The use of LNPs, however, needs to be further investigated to realize their potential in the formulation of a gene or drug delivery system for PD.

## 5. Conclusions and Future Perspectives

PD remains a major concern in the health sector, with therapeutics remaining at a palliative stage. The reduction in PD-associated symptoms is important, but the collateral side effects significantly impact the quality of the patient’s life. With the disease not being completely elucidated with regard to the mechanism of actions and causative agents, greater importance should be placed on unraveling these. Nanomedicine has provided a means of overcoming various challenges, with novel therapeutic drug and gene delivery systems providing a highly efficient mode of treatment that can potentially offer a cure for such a disease. An important role can be seen in RNA interference (RNAi) for silencing specific genetic-based mutations leading to PD, such the *LRRK2*, *PARK7*, *PINK1*, *PRKN*, and *SNCA* genes. The utilization of LNP-based vaccines to date have provided evidence that LNPs can be reasonably safe and efficient. These biocompatible LNPs can accommodate varied drug doses, be amenable to tissue-targeted delivery, and can be produced on a larger scale for commercial use. This has opened up new avenues to optimize LNP formulations for treating many disorders, including neurological disorders such as PD. The lack of studies using LNP formulations needs to be addressed, and basic research focusing on novel formulations directed to the brain needs to be encouraged. This will promote the optimization of LNP formulations for efficient brain targeting, which can be eventually translated to clinical settings. Paramount to brain targeting are the appending of suitable ligands such as angiopep-2 and transferrin to LNPs, which can improve their navigation of the BBB, and the use of polymers such as polyethylene glycol to ensure stability within the in vivo system. Importantly, the ability to design different LNP formulations for specific disorders may lead to a desired personalized form of treatment.

## Figures and Tables

**Table 1:** Genes commonly implicated in the onset of Parkinson’s disease.

Gene	Mechanism of Action	Dominant/Recessive	Ref.
***LRRK2***	Mutations in PD alter kinase and GTPase activities and promote substrate phosphorylation and autophosphorylation. The link to neuronal damage is still unclear.	Late-onset autosomal dominant familial PD	[28]
***PARK7***	Contains the *DJ-1* gene, which undergoes mutation, resulting in loss of gene expression. The mechanism of action is not elucidated, but mouse models show that the *DJ-1* gene may act as a neuroprotective redox sensor.	Autosomal recessive familial PD	[29]
***PINK1***	Has a regulatory role in the mitochondria, with damaged mitochondria undergoing mitophagy. Mitochondrial depolarization activates *PINK1* and causes phosphorylation of ubiquitin at Serine65 (Ser65). High-affinity binding to the E3 ligase ubiquitin (Parkin) primes it for phosphorylation by *hPINK1* at an identical Ser65 residue residing in the N-terminal ubiquitin-like domain. The E3 ligase activity is stimulated, resulting in substrates at the outer mitochondrial membrane undergoing ubiquitylation. Direct neuronal damage is still unclear.	Early onset recessive familial PD	[30]
***PRKN***	Encodes RBR E3 ubiquitin–protein ligases. Mutation results in the loss of this activity, leading to protein accumulation, mitophagy, and mitochondrial dysfunction. *PRKN* gene is named due to the “stereotypical” phenotypic outcomes.	Autosomal recessive juvenile PD (AR-JP)	[31]
***SNCA***	Integral in many cellular pathways, including protein degradation, membrane interactions, dopamine release and transport regulation, maintenance of synaptic vesicle supply, autophagy–lysosome pathway, and mitochondrial dysfunction.	Autosomal dominant PD	[32]

**Table 2:** The three broad classes of nanoparticles currently used in nanomedicine.

	Inorganic Nanoparticles	Organic Nanoparticles	Carbon-Based Nanoparticles
Examples	Quantum dots, metal oxide nanoparticles, metallic nanoparticles, mesoporous silica, bimetallic, and magnetic nanoparticles.	Solid lipid nanoparticles, micelles, liposomes, nanoemulsions, and polymeric nanoparticles.	Carbon nanotubes, fullerenes, graphene oxide, and nanodiamonds.
General Structure	Can contain core/shell structure from inorganic materials. Differ from their bulk material. 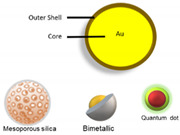	Generally comprise surfactants, cosolvents, and cosurfactants of organic nature. Lipid nanoparticles commonly contain phospholipids. 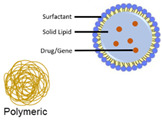	Includes sp^2^-hybridized carbon atoms. Have different shapes depending on the arrangement of the hexagonal lattice. 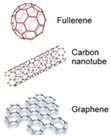
Properties	Facile synthesis. Provides a large surface area for large biomolecules. Tunable shapes and sizes [45].	Ease of preparation from biodegradable polymers. High stability in biological fluids and during storage [46].	Large surface area, high adsorption capacity, chemical inertness, thermal stability, and conductivity. Ideal for electrochemical detection [47].

**Table 3:** Some surface modifications to NPs for targeting the brain and increasing adsorption.

Ligand	Favorable Properties	Ref.
**Transferrin**	Transferrin receptors (TfR) are highly expressed in the BCECs and are thus commonly used targeting ligands. They promote efficient accumulation of therapeutics in the brain.	[71]
**Lactoferrin**	Lactoferrin, a glycoprotein present in the brain, acts as a receptor at the BBB. This approach has been identified to enhance the pharmacological properties of drugs. Furthermore, a positively charged group is exhibited upon binding, creating greater potential for NP entry.	[72,73]
**Glucose**	The BBB possesses glucose transporters (GLUTs) for active delivery of glucose into the brain to meet the high energy demand. NPs coated with glucose may be able to efficiently overcome the BBB via this transport system.	[74,75]
**Glutathione PEGylation**	PEGylated lipids with glutathione conjugates (G-Technology^®^) can pass through the BBB via the sodium-dependent transporter.	[76]
**Angiopep-2**	Has good transcytosis ability across the BBB. Can be conjugated to LNPs.	[8,77,78,79]

BCECs = brain capillary endothelial cells.

## Data Availability

Not applicable.
